# Effect of H-Terminated Surfaces on “Silicon-Vacancy” Fluorescence in High-Pressure Nanodiamonds

**DOI:** 10.3390/nano15241842

**Published:** 2025-12-05

**Authors:** D. G. Pasternak, A. A. Zhivopistsev, A. M. Romshin, O. S. Kudryavtsev, R. H. Bagramov, V. P. Filonenko, N. I. Kargin, I. I. Vlasov

**Affiliations:** 1 Prokhorov General Physics Institute of the Russian Academy of Sciences, 38 Vavilov Str., Moscow 119991, Russia; azh253@gmail.com (A.A.Z.); alex_31r@mail.ru (A.M.R.); leolegk@mail.ru (O.S.K.); 2 Vereshchagin Institute of High-Pressure Physics of the Russian Academy of Sciences, 14 Kaluzhskoe Shosse, Troitsk, Moscow 108840, Russiafilv@hppi.troitsk.ru (V.P.F.); 3 Institute of Nanoengineering in Electronics, Spintronics and Photonics, National Research Nuclear University MEPhI, 31 Kashirskoe Shosse, Moscow 115409, Russia

**Keywords:** nanodiamond, high-pressure synthesis, silicon-vacancy center, fluorescence, H-terminated surface

## Abstract

A new generation of fluorescent diamond nanoparticles synthesized from hydrocarbons at high pressure appears to be promising for the design of efficient single-photon diamond sources and nanometer-sized optical sensors. A characteristic feature of such nanodiamonds (NDs) is the termination of their surface with hydrogen. This hydrogen induces the formation of free holes at the diamond surface, thereby affecting the charge state of nearby fluorescent centers. In this study, the effect of the H-terminated ND surface on negatively charged silicon-vacancy (SiV^−^) fluorescence as a function of the ND size was investigated. Raman, photoluminescence and scanning electron microscopy techniques were used to characterize the NDs. Diamond nanoparticles of various sizes in the 50–300 nm range were analyzed before and after H desorption from their surface. It was shown that a significant increase in SiV^−^ fluorescence (>50%) upon hydrogen removal starts for particles smaller than 100 nm. The effective thickness of the diamond surface layer, within which charge neutralization of SiV^−^ centers occurs under the hydrogen influence, was determined to be 6 nm.

## 1. Introduction

A new generation of fluorescent nanodiamonds (NDs) synthesized from various hydrocarbons at high pressure and high temperature (HPHT) is attracting increasing attention from researchers developing single-photon sources (SPSs) and optical nanosensors. The synthesis of such diamond nanoparticles is typically carried out at a pressure of 7–9 GPa in the temperature range of 1000–1700 °C. The feasibility of creating efficient SPSs based on these HPHT NDs containing individual fluorescent nitrogen-vacancy (NV) [1,2,3], silicon-vacancy (SiV) [3,4,5], and germanium-vacancy (GeV) [6] centers has been demonstrated. NDs containing large ensembles of SiV centers have also been successfully employed as ultralocal temperature nanosensors in biological environments [7,8,9,10]. The possibility of the controlled formation of SiV centers in such NDs was recently shown [11]. For practical applications of fluorescent HPHT NDs, it is crucial to synthesize them with high structural and phase purity, free from residual graphitic inclusions, and to minimize the potential negative influence of the surface on their fluorescence intensity [12]. A characteristic feature of NDs synthesized from hydrocarbons is their hydrogen-terminated surface, which is rich in surface CH_x_ functional groups formed during the HPHT growth process [13]. Hydrogen termination induces the formation of a near-surface hole layer in diamond [14,15,16,17]. The most common fluorescent centers in diamond, negatively charged NV^−^, SiV^−^, and GeV^−^, can lose their excess electrons near the surface due to recombination with these holes, converting into neutral charge states. This results in the quenching of the centers’ fluorescence. For example, M.V. Hauf et al. [18] found that when changing the diamond surface termination from oxygen to hydrogen, previously stable NV^−^ centers, which were ion-implanted a few nanometers below the surface, converted into NV^0^. Petráková et al. [19] studied the influence of the hydrogenated surface of milled HPHT diamond nanoparticles doped with nitrogen on the intensity of NV^-^ fluorescence. They showed that NV^-^ fluorescence was completely quenched when the particle sizes were reduced to 20 nm. Stehlik et al. [20] demonstrated that the intensity of SiV^−^ fluorescence disappears in 8-nm-thick polycrystalline CVD diamond films doped with silicon and terminated with hydrogen.

The influence of a hydrogen-terminated surface on the fluorescence intensity of HPHT diamond nanoparticles synthesized from hydrocarbons has not yet been studied in detail. Here, we present a systematic investigation of this effect using electron-beam-induced hydrogen desorption from the surface of SiV^−^-fluorescent HPHT NDs, focusing on the size-dependent SiV^−^ emission.

## 2. Materials and Methods

The SiV-fluorescent NDs under study were synthesized by HPHT technique from a mixture of adamantane (C_10_H_16_, Sigma-Aldrich, St. Louis, MO, USA, 99% purity), detonation NDs (DND, Adamas Nanotechnologies Inc., Raleigh, NC, USA, average sizes 3–4 nm), containing ≈1% nitrogen impurity, and tetrakis(trimethylsilyl)silane (C_12_H_36_Si_5_, Sigma-Aldrich, St. Louis, MO, USA, >97%). The mixture was compressed to ≈7.5 GPa in a high-pressure chamber of the “toroid” type [21] and heated to 1350–1450 °C for 20 s. The adamantane/DND weight ratio was 25:1, and the Si/C atomic ratio was 1% in the initial growth mixture. A more detailed description of the HPHT synthesis procedure was given elsewhere [11].

Additionally, an ND sample was synthesized from a pure chloroadamantane (C_10_H_15_Cl, Sigma-Aldrich, St. Louis, MO, USA, 98%) under ≈7.5 GPa, ≈1350 °C. The sample was used to confirm effective hydrogen desorption from the diamond surface under electron irradiation. For this purpose, the intensities of CH_x_ vibrational modes in the Raman spectra of the sample were measured before and after electron irradiation. Unlike the SiV-containing sample, this “reference” sample facilitates the detection of the Raman CH_x_ band due to the small average size of its diamond particles (<50 nm) and the absence of fluorescence lines associated with donor-acceptor recombination [22], which could hinder observation of the CH_x_ Raman band.

A pre-characterization of the synthesized ND sample in a Scanning Electron Microscope (SEM, JEOL JSM-7001F, JEOL Ltd., Tokyo, Japan) revealed the ND size distribution predominantly in the range 50–300 nm for the main sample, and 30–50 nm for the “reference” sample. We employed an electron beam of the same microscope to remove surface-bound hydrogen from ND surfaces.

Micro-Raman spectroscopy and confocal laser reflectance imaging were performed using the multifunctional microscope (NTEGRA Spectra II, NT-MDT LLC, Zelenograd, Moscow, Russia). For the Raman measurements a continuous-wave 532-nm laser was used. The spectral resolution was ≈0.5 cm^−1^. Each Raman spectrum was accumulated over 180 s. During spectral acquisitions, the laser power at the sample was kept below 0.1 mW to avoid laser-induced ND heating. The confocal pinhole and objective optics conferred a diffraction-limited lateral spatial resolution of approximately 200 nm for both Raman spectrum and reflectance imaging. The confocal laser reflectance maps were recorded by a scanning the sample, which yielded high-contrast optical images of the NDs on a silicon substrate.

**Figure 1:**
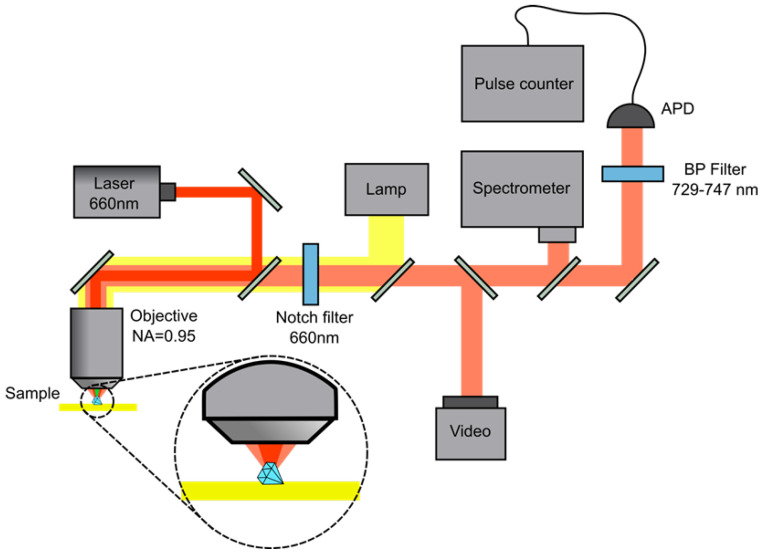
A schematic diagram of the custom-built confocal microscope used for the PL characterization of the ND samples.

A custom-built confocal microscope was used for the photoluminescence (PL) characterization of the synthesized SiV-NDs (Figure 1). It is equipped with a high-numerical-aperture objective NA = 0.95 (Olympus 100x, Olympus Corporation, Tokyo, Japan) and a piezoelectric translation stage (TRITOR 100, piezosystem jena GmbH, Jena, Germany), enabling three-dimensional fluorescence scanning with nanometer precision. Optical images of the ND were obtained using a high-sensitivity CMOS camera under white-light illumination. A continuous-wave 660 nm laser (GEM 660, Laser Quantum Ltd., Stockport, UK) served as the excitation source. A 660 ± 5 nm notch filter was placed into the registration channel to suppress back-scattered laser light. The PL spectra were recorded with an Optosky ATP5200 spectrometer (Optosky Photonics Inc., Xiamen, China): 600 grooves mm^−1^ grating, 100 µm entrance slit. Each PL spectrum was accumulated over an integration time of 10 sec with a laser power of 5 mW. For measurements of the SiV fluorescence saturation curves, the SiV- emission was detected by an avalanche photodiode (APD, SPCM-AQRH-14-FC, Excelitas Technologies Corp., Waltham, MA, USA) and processed with a photon counting module (Time Tagger 20, Swabian Instruments GmbH, Stuttgart, Germany). The SiV^−^ emission was spectrally isolated using a 738 ± 9 nm band-pass filter, ensuring that only the zero-phonon line (ZPL) region of the SiV^−^ fluorescence was collected.

## 3. Results and Discussion

### 3.1. The SiV Fluorescence Spectra of As-Grown HPHT Diamond Nanoparticles

**Figure 2:**
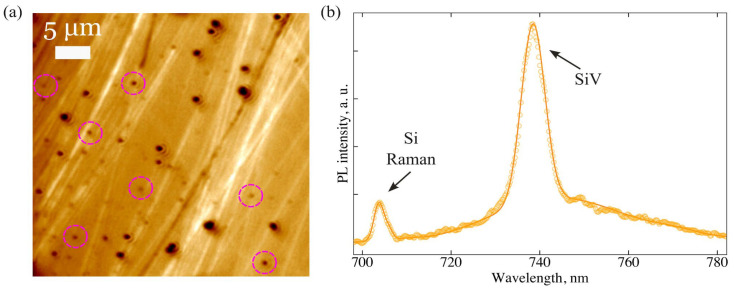
(**a**) Optical image of diamond nanoparticles distributed over a silicon substrate. Lilac dotted circles indicate representative ND spots selected for PL analysis. (**b**) A typical PL spectrum from one of the ND spots recorded under 660-nm laser excitation shows SiV line at 738.5 nm and the line of second-order Raman scattering from a Si substrate.

To study SiV fluorescence, diamond nanoparticles were distributed on a silicon substrate. For this, a low-concentration alcohol suspension of NDs was drop-cast onto a Si substrate and then allowed to dry. Figure 2a shows a characteristic optical image of the ND distribution. The dark spots in the image are associated with clustered or single ND particles. For PL analysis a large number (~100) of smallest spots were selected. Figure 2b shows a typical PL spectrum from one of the spots, where the SiV line at 738.5 nm is observed along with the second-order Raman scattering line from the Si substrate. The diamond Raman peak near 724 nm does not appear in the spectrum, which indicates a small (<150 nm) size of the diamond cluster or particle.

The dependence of the integrated SiV fluorescence intensity on the excitation laser power was determined in the 738 ± 9 nm spectral range using an APD (see Section 2). For each measured spot, the PL signal was optimized by performing three-dimensional (3D) scanning of the sample to achieve the maximum fluorescence intensity. The measured dependencies are presented below (see Section 3.3).

### 3.2. Surface Hydrogen Desorption and Size Characterization of Diamond Nanoparticles

The SEM electron beam was used to remove hydrogen from the surface of the NDs under study. Electron beam irradiation was first employed to remove hydrogen from bulk diamond surfaces [23]. Recently, we successfully applied this approach to desorb hydrogen from the surface of HPHT NDs [22].

**Figure 3:**
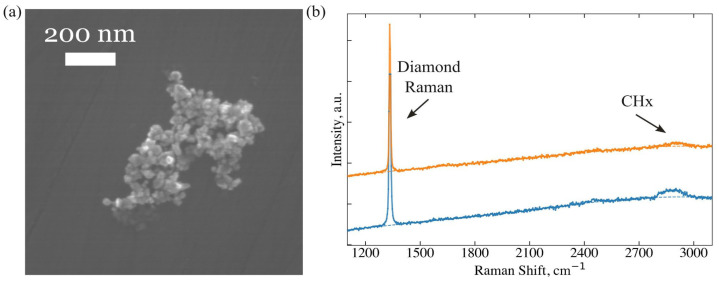
(**a**) The SEM image of a representative cluster of “reference” NDs (30–50 nm grain size) selected for hydrogen removal by an electron beam. (**b**) Raman spectra of the same ND cluster recorded before (blue) and after (orange) electron-beam treatment (laser radiation wavelength is 532 nm, and laser power ≈100 μW).

First, the hydrogen removal efficiency was confirmed using a “reference” ND sample (see Section 2). Raman spectra were recorded for a large cluster of the NDs before and after electron beam (20 keV) irradiation (Figure 3). After a 1-min electron beam exposure, the integral intensity of the CH_x_ vibrational bands (2800–2950 cm^−1^) decreased fivefold while the diamond Raman peak at 1332.1 cm^−1^ remains unchanged.

**Figure 4:**
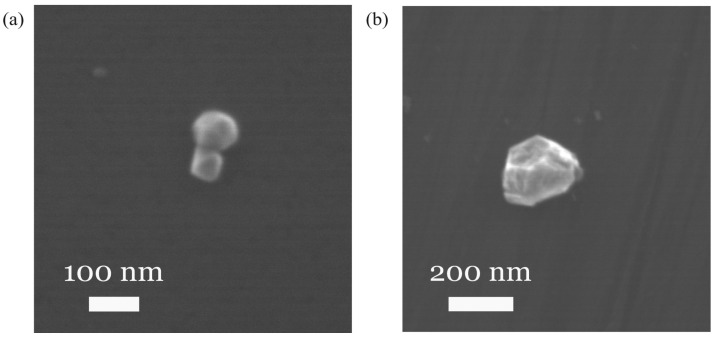
The characteristic SEM images of SiV-fluorescent NDs. Their estimated sizes are 70 nm (**a**) and 270 nm (**b**).

Next, the SiV^−^-containing NDs were re-examined. All small spots previously examined for SiV fluorescence (see Section 3.1) were irradiated using SEM electron beam to desorb hydrogen. From these, spots consisting of a single or a small number of similarly sized grains were selected. A total of 14 diamond clusters (particles) with particle sizes ranging from 50 to 300 nm were selected. The characteristic SEM images of selected NDs with sizes of approximately 70 nm and 250 nm are shown in Figure 4.

Their sizes were estimated in the spherical shape approximation. For each SEM image of the diamond nanoparticles, we determined the diameter of the largest circle inscribed in the particle contour using Fiji software [24].

### 3.3. Comparative Analysis of SiV Fluorescence in Diamond Nanoparticles Before and After Hydrogen Desorption

**Figure 5:**
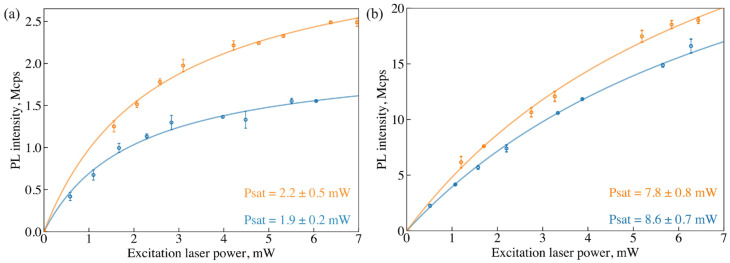
Comparative curves of SiV fluorescence saturation measured for different NDs before (blue) and after (orange) hydrogen desorption. Data for NDs with the diameters of 70 nm (**a**) and 250 nm (**b**). The plots show the fluorescence count rate I of SiV PL as a function of laser excitation power P (λ_exc_ = 660 nm). The circles are the background-corrected experimental data, and the solid lines fit to the saturation function $I(P)=I_{\infty }\cdot (\frac{P}{P+P_{sat}})$, from which the maximum count rate $I_{\infty }$ and the saturation power $P_{sat}$ are obtained. The uncertainties of the fitted parameters were estimated as the square roots of the diagonal elements of the covariance matrix returned by the fitting routine. The error bars represent pointwise deviations of the experimental data from the fitted saturation curve.

For the 14 spots selected in the previous step of the study, the SiV fluorescence spectra were recorded again, and the dependencies of the integrated SiV fluorescence intensity on the laser excitation power (saturation curves) were determined. An increase in SiV^−^ fluorescence intensities was observed for all examined NDs after hydrogen removal. Characteristic curves of the SiV fluorescence saturation measured before (blue) and after (orange) hydrogen desorption are shown in Figure 5.

The saturation powers ($P_{sat}$) of SiV fluorescence obtained from these dependencies were found to coincide within the error limits before and after the electron-beam treatment. This indicates that the excitation efficiency of SiV fluorescence remains essentially unchanged, and that the observed increase in its intensity after hydrogen removal is primarily due to an increase in the number of active SiV^−^ centers within the NDs.

**Figure 6:**
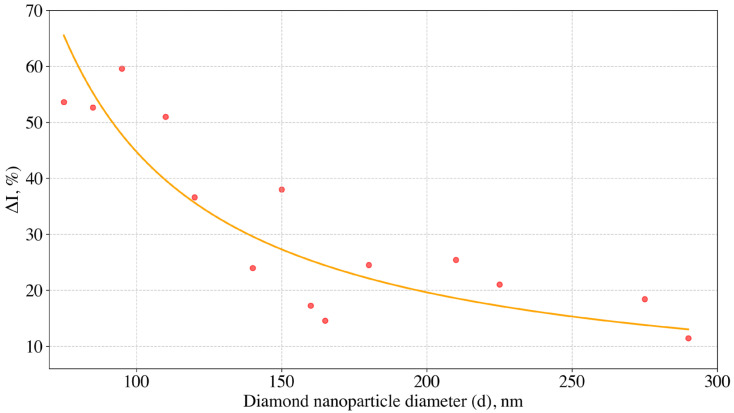
The dependence of the relative increase in SiV fluorescence intensity ΔI(d) on the ND diameter d upon hydrogen removal from the surface of HPHT NDs (red circles). The solid orange line is a fit to the model $\Delta I(d)=100\%\cdot ({\left(\frac{d}{d-2W}\right)}^{3}-1)$, (see details in the text) The SiV fluorescence intensities were measured at λ_exc_ = 660 nm, P_exc_ ≈ 5 mW.

The dependence of relative increase in the SiV intensity (ΔI) on the particle size (d) is presented in Figure 6 and in Table 1. This increase is defined as the dimensionless value

$$\Delta I=100\%\;*\;(\frac{I_{after}}{I_{before}}-1),$$

here $I_{after}$ and $I_{before}$ are the integrated SiV fluorescence intensities after and before hydrogen desorption, respectively.

As can be seen, the influence of the H-terminated surface on the SiV fluorescence becomes noticeable even for NDs with sizes around 300 nm, while a significant enhancement (>50%) of SiV emission upon hydrogen removal is observed for particles smaller than ≈100 nm. The obtained dependence (Figure 6) makes it possible to estimate the effective thickness of the near-surface diamond layer where the SiV^−^ fluorescence is suppressed under the influence of surface hydrogen. For simplicity, we assume that the diamond nanoparticles are spherical with a diameter d, the SiV-quenching layer has a uniform thickness W along the entire surface, and the SiV centers are homogeneously distributed throughout the diamond volume. Upon hydrogen removal, all SiV centers within the diamond volume are considered to become optically active. Under these assumptions, the experimental dependence of the relative fluorescence increase ΔI(d) can be approximated by the expression

$$\Delta I(d)=100\%\cdot ({\left(\frac{d}{d-2W}\right)}^{3}-1),$$

where W is an effective thickness of the hydrogen-induced quenching layer, used for data fitting in Figure 6. From this model, we derive a value of W=6.0±0.4 nm.

**Figure 7:**
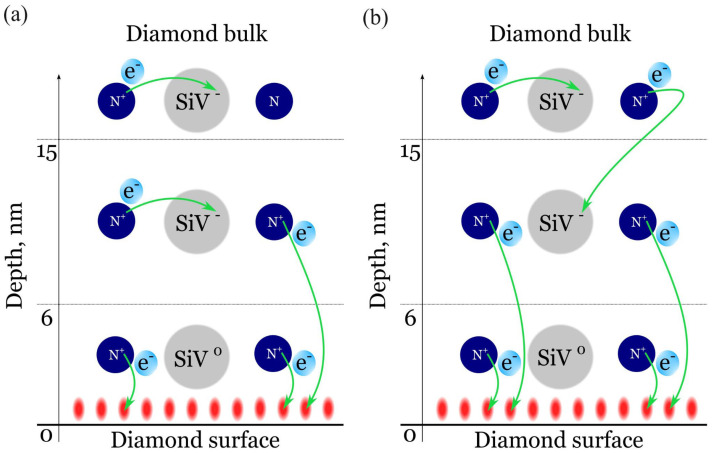
Schematic illustration of possible interactions of donor nitrogen with SiV and near-surface holes next to H-terminated diamond surface. Within 6 nm from the surface N donates electrons predominantly to the surface holes, and SiV^0^ remains uncharged. At depths within 6–15 nm SiV^0^ can transfer to SiV- in two ways: (**a**) N donates an electron to neighboring SiV^0^ with higher probability than to surface holes; (**b**) SiV^0^ is negatively charged by N located more than 15 nm from the surface.

The main source of electrons required for the formation of negatively charged SiV centers (SiV^−^) is the donor-like substitutional nitrogen impurity present in the diamond lattice [25,26,27,28,29]. Near the hydrogen-terminated diamond surface, two competing compensation channels for these nitrogen donors arise: (i) a thin (≈1 nm) layer of free holes, and (ii) neutral SiV^0^ centers. According to J. Ristein et al. [30], the holes recombine with electrons from nitrogen donors located up to ≈15 nm beneath the diamond surface. If this recombination channel is more efficient than the donation of an electron from nitrogen to the nearby SiV^0^, then SiV^−^ cannot form within this ≈15 nm region. Comparing the results of the present work with those of Ristein et al. [30], we find that the depth of the region where nitrogen donors are compensated by surface holes is approximately 2.5 times greater than the depth (≈6 nm) at which SiV centers remain neutral under hydrogen termination. This discrepancy can be rationalized by assuming that beyond 6 nm from the surface, the probability of nitrogen compensation by neighboring SiV^0^ is higher than by surface holes (Figure 7a). Alternatively, nitrogen located deeper than 15 nm from the surface can charge SiV^0^ located in the 6–15 nm depth range from the surface (Figure 7b).

For the comparison, we refer to previously reported results on the influence of surface hydrogen on the charge states of SiV and NV centers in diamond nanostructures of different origins. In particular, Stehlik et al. [20] found that the intensity of SiV fluorescence disappears in polycrystalline CVD diamond films doped with silicon and terminated with hydrogen for film thicknesses of less than 8 nm. Considering the 2-nm nucleation layer, the quenching of SiV emission occurs in the near-surface region with a thickness of about 6 nm. Thus, the estimates of the thickness of H-terminated layers suppressing SiV- fluorescence in diamond films and nanoparticles are in good agreement with each other. Petráková et al. [19] showed that NV- fluorescence was completely quenched in hydrogenated HPHT diamond nanoparticles of 20 nm in size, corresponding to an effective quenching layer thickness of 10 nm. These results indicate that hydrogen terminating a diamond surface exerts a stronger influence on the charge state of NV than on SiV centers.

## 4. Conclusions

The effect of hydrogen-terminated surfaces on the SiV^−^ fluorescence intensity in HPHT NDs synthesized from hydrocarbons and doped with silicon was studied. SiV-fluorescent diamond nanoparticles with sizes ranging from 50 to 300 nm were analyzed before and after hydrogen removal from their surfaces. It was found that the influence of surface hydrogen termination on the SiV fluorescence becomes noticeable even for NDs with diameters around 300 nm, while a significant enhancement of fluorescence (>50%) upon hydrogen desorption occurs for particles smaller than ≈100 nm. From the measured dependence of SiV fluorescence intensity on particle size, we determined the effective thickness of the near-surface layer in which the charge neutralization of SiV^−^ centers takes place to be approximately 6 nm.

A new generation of HPHT NDs synthesized from hydrocarbons is the promising nanomaterial for developing high-performance single photon sources and nanoscale optical sensors based on SiV^−^ emission. These NDs exhibit high crystalline quality, and under optimized synthesis conditions, they are essentially devoid of graphitic inclusions [13]. An intrinsic limitation for the practical application of such as-grown diamond nanoparticles is the presence of hydrogen on their surface, which induces suppression of SiV fluorescence near the surface. To overcome this drawback, a post-growth surface treatment is required to maximize the optical performance of SiV-based quantum emitters and sensors.

## Figures and Tables

**Table 1:** The dependence of $\Delta I,{\;I}_{after}$ and $I_{before}$ on the ND diameter *d*.

*d*, nm	75	85	95	110	120	140	150	160	165	180	210	225	275	290
*I_before_*, kcps	3.2	3.5	359	5.1	86	206	196	235	245	538	1337	2683	3524	1756
*I_after_*, kcps	4.9	5.3	570	7.7	118	255	271	276	281	675	1687	3256	4173	1948
*∆I*, %	53	51	59	51	37	24	38	17	15	25	26	21	18	11

## Data Availability

Data are contained within the article.
